# Prospects for the application of artificial intelligence in geriatrics

**DOI:** 10.1515/jtim-2024-0034

**Published:** 2025-01-10

**Authors:** Li Zhang, Jing Li

**Affiliations:** Department of Geriatrics, Xuanwu Hospital Capital Medical University, National Clinical Research Center for Geriatric Diseases, Beijing, China

## Introduction

Aging is a major challenge faced by world, which has profound impacts on society. Recent years, artificial intelligence (AI) technology has developed rapidly. AI refers to the behavior that a computer can do intelligent behavior with minimal human intervention,^[1]^ which has shown great potential in health care which can improve patient and clinical team outcomes, reduce costs, and influence population health.^[2]^

## Application of AI in medicine

**Figure 1:**
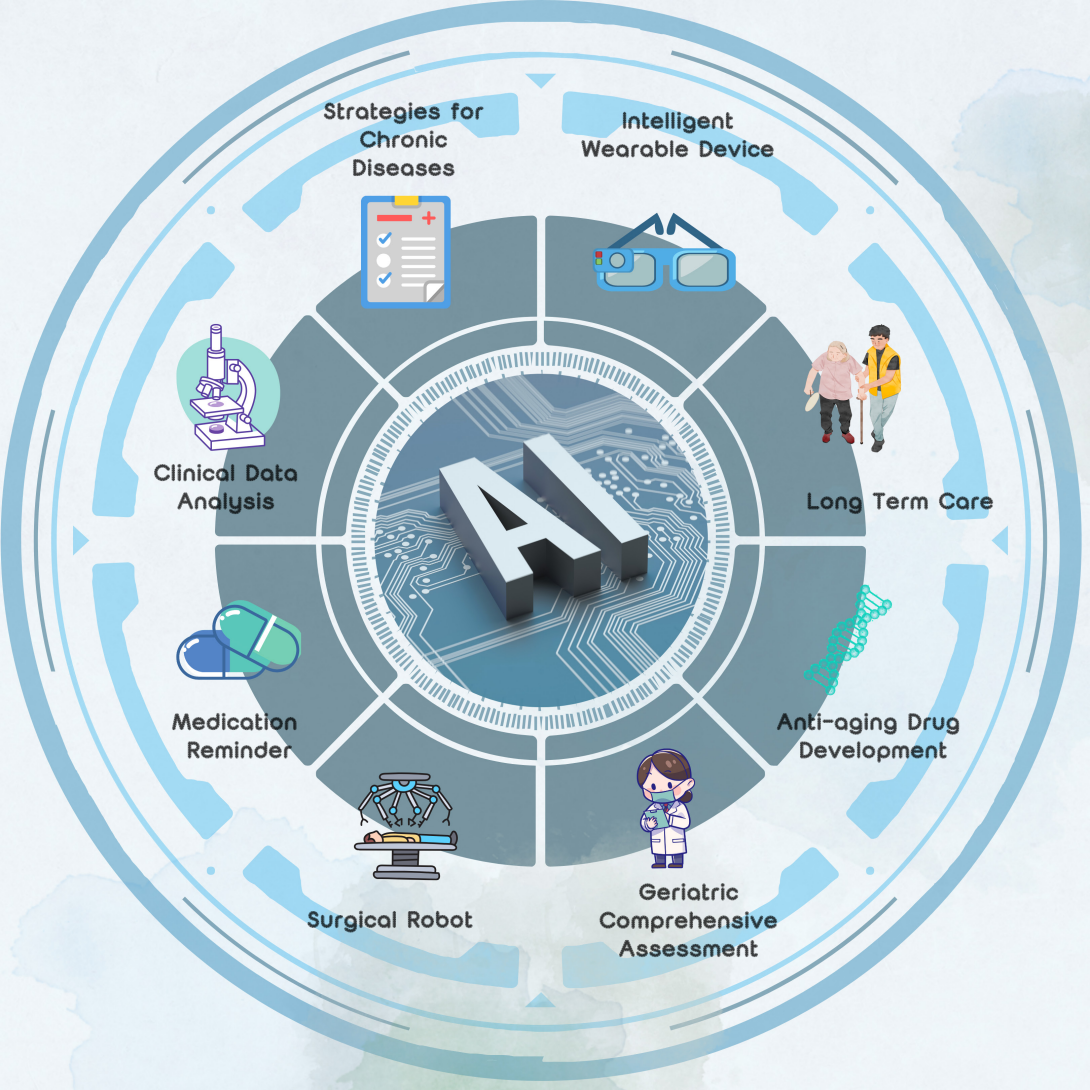
Application of Artificial Intelligence in Geriatrics.

AI has applications across various fields of medicine (Figure 1). Generative AI methods can create designs, such as small-molecule drugs and proteins, by analysis diverse data modalities, including images and sequences.^[3]^ The da Vinci surgical robot^[1]^ has been widely applied in complex surgical procedures, such as thoracic surgery, neurosurgery, gynecology, which safety has been validated in clinical practice. The earliest AI systems reanalyzed large amounts of clinical data and assisted in clinical diagnosis. Jha and Topol urged radiologists to adopt AI technologies when analysis diagnostic images that contain vast data information.^[4]^ However, up until now, AI can enhance the efficiency of clinical workers and assist doctors in diagnosis and treatment, but it cannot completely replace the work of doctors.

## Management of chronic diseases in older adults by AI

AI has not only been widely applied across various medical specialties but also shows promising prospects in the field of geriatric medicine. Older adults often face chronic diseases and even multimorbidity. Patients’ physiological parameters, laboratory test results, and imaging data can analyzed by AI and help doctors promptly identify potential health issues. AI not only aids in disease identification but also optimizes treatment plans by analyzing individual patient data. Dementia is very common in older adults, which often difficult to diagnose due to its subtle early symptoms. AI has already been applied in monitoring biological markers ^[5]^ for Alzheimer’s disease and in automated imaging analysis.^[6]^ Perhaps in the future, AI may be able to automatically collect everyone’s living habits, genetic status and examination result, *etc*., to provide early warnings of diseases for older adults.

## Management of geriatric syndromes by AI

Geriatric Syndromes (GS) refer to the multifactorial health conditions that occur when the cumulative effects of functional disorders of multiple systems make an individual vulnerable to face the challenges of situations.^[7]^ Geriatric syndromes are risk factors for adverse outcomes such as disability and morbidity in older adults. AI technology excels in processing complex multidimensional data, which could comprehensively assess and manage geriatric syndromes by integrating multidimensional data. Frailty, as one of the geriatric syndromes, significantly disrupts the lives of elderly individuals. However, it is challenging to conduct frailty screening for every older adult in daily life. In the early screening of frailty syndrome among older adults, AI can leverage electronic health records (EHR), wearable device data, and imaging data to accurately identify high-risk individuals.^[8]^ Older adults often require continuous monitoring, which the traditional healthcare system struggles to provide. AI-powered intelligent devices, such as wearables and home health monitoring systems, can record physiological data from older adults in real time and automatically alert healthcare professionals upon detecting abnormalities. This greatly facilitates the long-term management of frailty.

## Application of AI in older adults’ care

Generative AI represents a major revolution in AI capabilities and offers great potential for improving care. AI has been used in older adults care, alleviating issues related to staff shortages and uneven resource allocation.^[9]^ AI can provide convenience for older adults and caregivers to communicate without face-to-face, which can reduce the number of hospital visits for them. At present, AI can provide intelligent medication reminders, and in the future, intelligent robots may replace the current caregivers, and robots can assist the older adults complete the difficulties encountered in daily life, such as bathing, turning over, feeding and so on. However, there are also viewpoints that people’s excessive reliance on AI robots for care may lead to a deterioration in the skills of provider, and making them unable to provide proper care when faced with issues or malfunctions with the robots.

## Application of AI in anti-aging drug research

Drug development is an important part of anti-aging research. AI can analyze existing drugs, compounds, and clinical data to predict which drugs may be effective in combating aging and quickly screen potential candidates. By accelerating the drug development and testing process, AI can reduce the time and cost associated with traditional drug research. For example, the governing key factors (*e.g*., organic loading rate, dissolved oxygen level, nutrients, temperature, pH, solids concentration, toxicity, *etc*.), as well as microbial community structure, intracellular metabolic interaction, and enzymatic activity, collectively influence the overall performance.^[10]^ Additionally, AI can help determine the optimal dosage and drug combinations, minimizing side effects and enhancing efficacy, thereby helping elderly individuals better manage age-related diseases.

## Application of AI in rehabilitation

In addition to illnesses, older adults often experience the decline in physical function. AI could enable interdisciplinary collaboration by capturing movement data from older adults—such as gait analysis and joint flexibility measurements—along with their overall physical condition. AI could integrate expertise from physical therapists, nutritionists, and psychologists to develop more precise personalized nutrition plans and training programs, enhancing the interactivity and enjoyment of exercises while ensuring real-time safety monitoring. Additionally, AI could analyze indicators such as voice tone, facial expressions, and heart rate to assess emotional changes during rehabilitation. Based on this, it could provide dynamic psychological support and companionship, thereby improving adherence to rehabilitation training.

## Possible problems with AI

Despite the exciting prospects of AI in the field of anti-aging, it also comes with certain ethical and social issues. At present, traditional family care is more common for the older adults. Most older adults may have a low acceptance of new technologies, which can limit the application of AI in caregiving. Moreover, a large amount of personal information needs to be analyzed by AI, and concerns about privacy are a growing issue. How to effectively protect patients’ privacy and prevent data breaches and misuse is a topic that requires further exploration. Currently, AI primarily assists humans by quickly processing and analyzing large datasets. In the near future, as AI’s logical thinking abilities continue to improve, there is a concern about whether AI could eventually replace humans or even pose a threat to humanity with its advanced cognitive capabilities.

In summary, AI has great potential in geriatric medicine to enhance the efficiency and personalization of healthcare services. However, careful consideration of ethical issues, privacy concerns, and interpersonal relationships is necessary during its implementation to ensure comprehensive and compassionate care for older adults.
